# Low-cost IoT based system for lake water quality monitoring

**DOI:** 10.1371/journal.pone.0299089

**Published:** 2024-03-28

**Authors:** Kartikay Lal, Sanoj Menon, Frazer Noble, Khalid Mahmood Arif

**Affiliations:** Department of Mechanical and Electrical Engineering, Massey University, Auckland, New Zealand; Lahore Garrison University, PAKISTAN

## Abstract

Water quality monitoring is a critical process in maintaining the well-being of aquatic ecosystems and ensuring growth of the surrounding environment. Clean water supports and maintains the health, livelihoods, and ecological balance of the ecosystem as a whole. Regular assessment of water quality is essential to ensure clean and reliable water is available to everyone. This requires regular measurement of pollutants or contaminants in water that can be monitored in real-time. Hence, this research showcases a system that consists of low-cost sensors used to measure five basic parameters of water quality that are: turbidity, total dissolved solids, temperature, pH, and dissolved oxygen. The system incorporates electronics and IoT technology that are powered by a solar charged lead acid battery. The data gathered from the sensors was stored locally on a micro-SD card with live updates that could be viewed on a mobile device when in proximity to the system. Data was gathered from three different bodies of water over a span of three weeks, precisely during the seasonal transition from autumn to winter. We adopted a water sampling technique since our low-cost sensors were not designed for continuous submersion. The results show that the temperature drops gradually during this period and an inversely proportional relationship between pH and temperature could be observed. The concentration of total dissolved solids decreased during rainy periods with a variation in turbidity. The deployed system was robust and autonomous that effectively monitored the quality of water in real-time with scope of adding more sensors and employing Industry 4.0 paradigm to predict variations in water quality.

## Introduction

**Fig 1:**
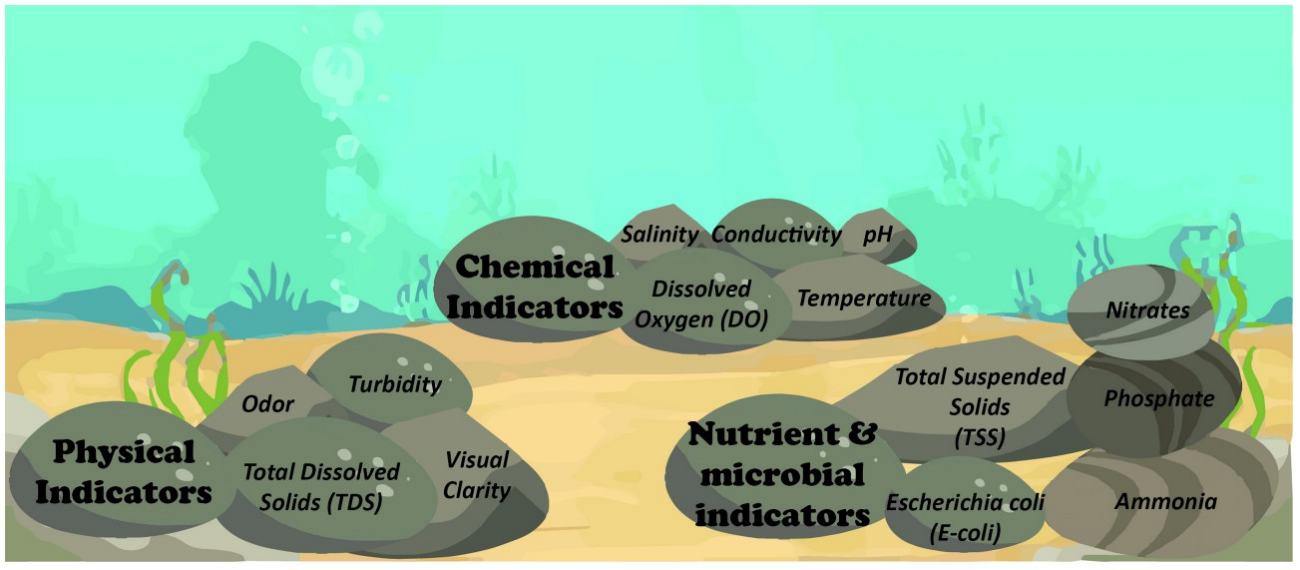
Physical, chemical, nutrient and microbial indicators of water quality.

The determination of water quality is desirable for a wide range of reasons. Firstly, water quality has a significant impact on aquatic life, plant growth, and human health. There are key indicators that can be used to clearly define water quality that are primarily broken into four categories: 1) biological indicators; 2) chemical indicators; 3) physical indicators; and 4) recreational indicators. Some of the indicators that are within these categories are: water conductivity, salinity, acidification, wastewater chemicals like E-coli and ammoniacal nitrogen, chlorophyll, water clarity, sediment enzymes, sediment mercury, algal toxins, Nitrogen, Phosphorus, dissolved Oxygen, Cyanobacteria, Atrazine, and Oxidation Reduction Potential (ORP) [1, 2]. Fig 1 depicts a diverse range of indicators that provide information about different aspects of water quality. The physico-chemical attributes, such as dissolved oxygen and temperature have a large impact on aquatic life, the nutrient elements, such as nitrogen and phosphorus, greatly benefit plant growth; whereas, faecal microbial contaminants, such as E-coli, can have an adverse effect on human health [3]. Freshwater sources are facing a worldwide scarcity due to significant human utilization and pollution. Only 0.6% of the world’s water is considered freshwater, and currently, 85% of this small percentage is used in agriculture.

To determine the condition of a body of water, aforementioned indicators or parameters have been established to collectively determine the water quality, from which five are considered to be the most fundamental parameters that include pH (power of Hydrogen), turbidity, Dissolved Oxygen (DO), Total Dissolved Solids (TDS) and temperature. These fundamental parameters are important to learn from the natural processes in the environment and determine human impacts on the ecosystem [4]. These parameters also provide valuable information about the physical, chemical, and biological characteristics of water while ensuring environmental standards are being met [5]. For instance, pH refers to the level of acidity or alkalinity of water, which is measured by the concentration of hydrogen ions present, providing a numerical pH value. pH scale varies from 0 to 14. A value of 7 denotes a neutral state, while values above and below this state determine the alkaline or acidic nature of water. Turbidity refers to the degree of cloudiness of water, which arises from solids or materials suspended in water. For example, healthy water for drinking should have turbidity less than one Nephelometric Turbidity Unit (NTU). Temperature refers to how hot or cold water is, measured in degree Celsius. Temperature is associated with affecting the physical and biological processes in aquatic environments. DO refers to the quantity of oxygen dissolved in water which determines the quality of life led by aquatic organisms which greatly depends on temperature. For instance, low dissolved oxygen can flourish aquatic beings such as fish. Low dissolved oxygen can arise from pollution or poor water circulation. Warm water holds less DO than cold water, and some compounds are more toxic to aquatic life at higher temperatures [6]. Furthermore, TDS refers to the amount of suspended particles in water. This can be in the form of silt, clay, organic matter, or other pollutants. Table 1 indicates water quality parameters that are relevant to this study and the standards set by the World Health Organization (WHO) that defines typical values for ensuring good water quality.

**Table 1:** Water quality parameters with WHO standard of clean water.

Parameter	WHO standard	Units
pH	7–8.5	
Turbidity	1–5	NTU
Dissolved Oxygen (DO)	5–6	mg/L
Total Dissolved Solids (TDS)	500	ppm
Temperature	15	°C

Due to natural and anthropogenic release of contaminants into water, need for instantaneous measurement of water quality has become imperative. Cost-effective and real-time assessment of water quality over a certain period of time would allow end users such as government agencies and city council to access detailed evidence and trends that can support informed decisions toward managing the quality of water. With the advancement in sensing technologies and long range wireless communication networks, a low-cost water quality monitoring system can be developed to provide real-time data on condition of water quality [7]. This data can be remotely monitored and analyzed by the end users, which would be helpful in initiating any necessary interventions to keep water parameters within the required standard [8], as defined in Table 1.

Several laboratory-based methods are available that offer precise measurements with high sensitivity. Research in the field of water quality testing predominantly takes place using traditional sampling and laboratory methods that suffer from limitation. These drawbacks include monotonous process of sample collection which is time consuming and require a specialized facility with sophisticated laboratory equipment and trained personnel to operate [9]. This process can be very costly that also involves high overheads. Table 2 summarises strengths and weaknesses of laboratory-based and on-site testing methods.

**Table 2:** Advantages and disadvantages of on-site and lab-based water quality testing.

**On-Site Water Quality Monitoring**		
**Advantages**	**Disadvantages**	**Ref**
Real-time results	Offer limited Parameters	[14]
Cost-effective	Lower accuracy	
Rapid-response	Regular calibration of sensors maybe required	
Automated data analysis	Complex analysis maybe limited	
**Laboratory-Based Water Quality Testing**		
**Advantages**	**Disadvantages**	**Ref**
Comprehensive list of parameters can be included	Time consuming	[15]
Highly accurate	Costly	
Sensitive detection using specialized equipment	Delay in receiving results	

Ample research has taken place all over the world in studying the quality of water that largely involves sample collection and testing in a laboratory. Few studies have come to light that can provide regular updates on water quality, either through direct text messaging or cloud based monitoring but still rely on collection of sample and testing them on a system built with sensors and the IoT communications technologies. Most of the systems signal the user when any of the parameters are outside the preset thresholds.

## Related solutions

Studies around water quality assessment have been carried out by numerous researchers worldwide, utilizing low-cost sensors integrated into various systems such as buoys and custom-designed flotation devices. The systems not only monitor five basic parameters, but also measure Oxidation-Reduction Potential (ORP), Electrical Conductivity (EC), Total Suspended Solids (TSS), and more. Additionally, these studies leverage long-range communications and cloud services for effective data transmission and monitoring.

Demetillo et al. [10] developed two buoys and monitored water temperature, DO, and pH in a pre-programmed time interval. All sensors are connected to a microcontroller that stored information in a database. Zigbee modules were used for communication between the two nodes while a GSM module was used for sending text messages to the end-user. The information was displayed graphically on a web page that could be accessed through preregistered mobile phone for quick monitoring for only selected end-users. Similarly, Prasad et al. [11] built a system housed with four sensors namely temperature, pH, ORP and conductivity. The sensors, SD card module, and GSM module, interfaced to an Arduino Uno board that took readings from the sensors at interval of 15 minutes. Water quality was tested in tap water, creek, coast, and sea water samples. The data showed a relationship between temperature, pH and conductivity of the sample. Temperature was proportional to conductivity and inversely proportional to pH. The system was programmed to send a text message to the user if any of the parameters were detected beyond the water quality standard. Another study conducted by Rao et al. [12] demonstrated a continuous water quality monitoring system in a laboratory environment by incorporating low-cost sensors including temperature, light intensity, TDS, pH, salinity, DO, Electrical Conductivity (EC), and ORP. These parameters provided physio-chemical insights into the current condition of water and potentially identify sources of pollution. They collected samples at regular intervals for chemical analysis in the laboratory to ensure a healthy environment for aquatic life. Geetha et al. developed a system to monitor turbidity, temperature, conductivity, and pH level of water sources around their university. The sensors connected to a microcontroller with built-in Wi-Fi. The data was sent to the cloud and real-time sensor readings were displayed on an on-board LCD. A GSM module also sent text messages to user when the quality of water was detected outside predefined range. Similarly, Chowdury et al. [13] proposed a low-cost, power efficient system with 4 sensors namely pH, turbidity, temperature, and ORP sensor. They collected river sample and tested on the system that integrated an ESP-8266 Wi-Fi module and sensor data could be viewed on LCD screen mounted on a printed circuit board or a mobile phone. Pasika et al. [16] built a system by incorporating pH, turbidity, water level sensor, along with temperature and humidity for the surrounding atmosphere. Their system revolved around filling a tank with water procured from a metropolitan water supply, taking sensor readings every 10 seconds and then uploading to Thingspeak cloud service. Although their system is capable of providing real-time sensor data, it is not field-deployable.

**Fig 2:**
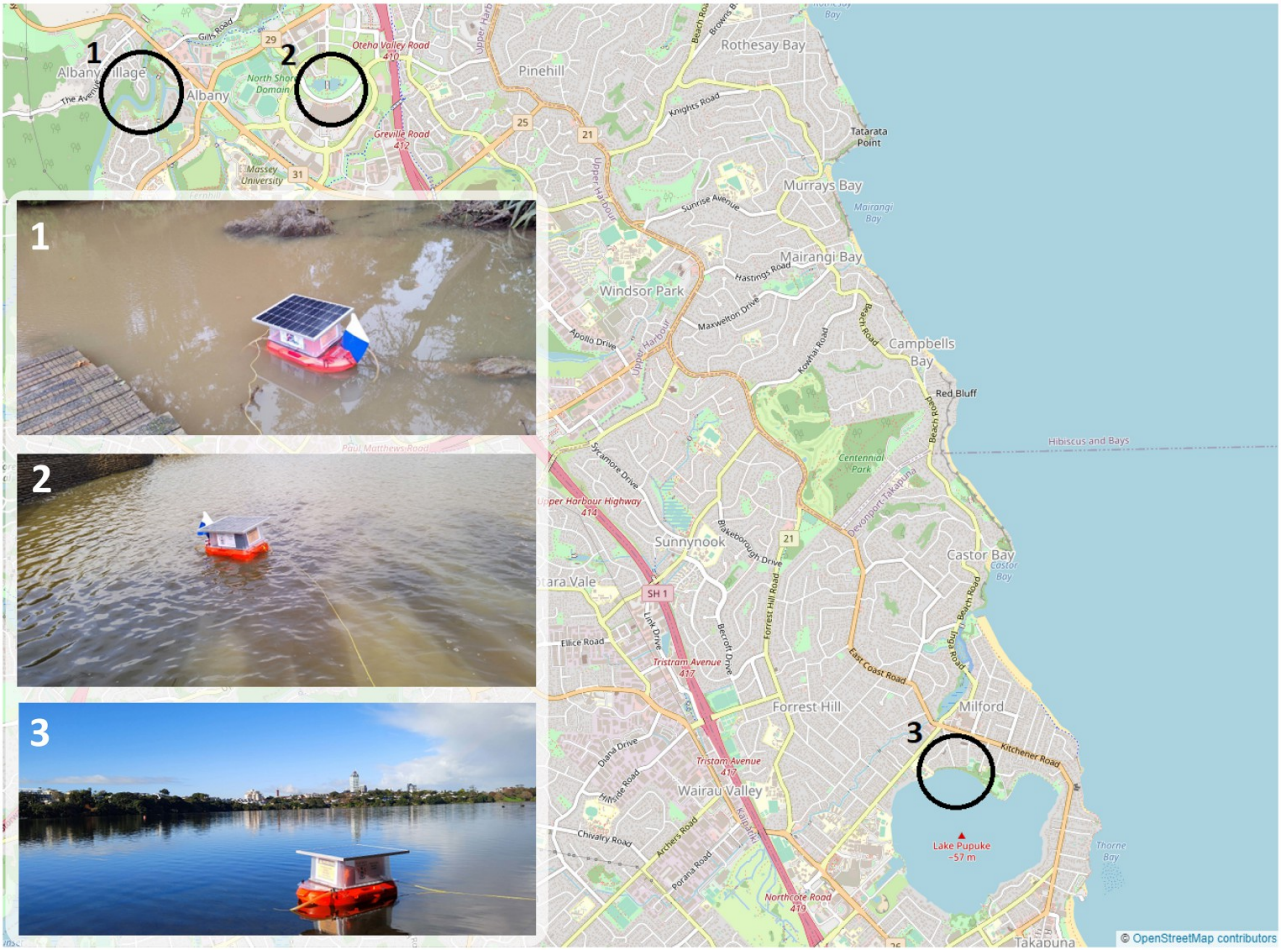
The labelled areas highlight three testing sites. 1. Lucas Creek, 2. Lake Albany, 3. Lake Pupuke.

Studies show that anthropogenic and natural activities have a big impact on the quality of water and its influence on the water quality parameters when it rains. There are systems that perform machine learning on the collected data to predict changes in water quality [17]. While these studies have provided informative results, current water monitoring systems still suffer from challenges with laborious sample collection. This kind of system would be incapable of establishing the quality of water in real-time and how that varies over a period of time. Hence, an in-situ real-time monitoring system was built that involved a flotation device, installed with five low-cost water quality sensors. The system was deployed in three different water bodies located in the suburb of Albany in Auckland, New Zealand. The locations are marked on the screenshot taken from OpenStreetMap as shown in Fig 2. Our objective was to measure water quality of freshwater sources and how it was influenced by weather conditions. Lakes are known to have freshwater that is ever-changing due to inflows and outflows [18]. Inflows occur from rain, overland runoff, and groundwater seepage while outflows are caused by seepage, into groundwater and evaporation. However, inflow and outflow occur at a very slow pace. Lakes are great habitats to study physio-chemical dynamics of ecosystem. Lakes are immobile bodies of water which means they preserve their natural characteristics and keep nutrients relatively stable without abrupt changes. To build a cost-effective water quality system, we utilized inexpensive sensors that require a stable environment for detection. Table 3 outlines common laboratory-based detection techniques for water quality parameters. To conduct the experiments, we chose lake water because water quality parameters are expected to be fairly constant with negligible variation as compared to a river or ocean, which was suitable for our low-cost sensors.

**Table 3:** Common laboratory-based detection techniques for fundamental water quality parameters.

Parameter	Measurement techniques	Ref
Temperature	Thermocouples	[19]
	Thermistors	
	Infrared (IR)	
	Resistance Temperature Detectors (RTDs)	
	Liquid glass tube thermometer	
	Digital Temperature probes*	
Dissolved Oxygen	Optical—luminescence quenching	[20]
	Winkler titration	
	Membrane electrode*	
Total Dissolved Solids	Conductivity meter*	[21]
	Gravimetric (filtration of residual solids)	
	Optical—light scattering and absorption	
Turbidity	Optical—light scattering*	[22, 23]
	Optical—laser diffraction	
	Optical—light absorption	
pH	Glass electrode method*	[24]
	pH test strip or litmus paper	
	Potentiometry	

### Water quality assessment models

Water quality assessment models are the criteria that grade the quality of any given body of water; this is typically done using a Water Quality Index (WQI) that is used to evaluate the overall water quality of a body of water that can be compared against city council approved index [25]. There are multiple water quality assessment models that are used throughout the world to evaluate different types of parameters, from chemical pollutants in water to micro-invertebrate contamination [26]. The water quality index is a score that is established by ranking water quality parameters according to their level of importance as defined by the water quality standards. Most countries have their own modelling systems that are used to determine the water quality of bodies of water. Two examples of WQIs used by governments today are the Canadian Council of Ministers for Environment (CCME) [27] and Aggregation Functions [28]. The CCME is primarily used in New Zealand and Canada while the use of aggregation functions has been popularised in the United States of America (USA). The CCME WQI requires that there be a minimum of four water quality parameters being measured, but puts no restriction on what those parameters might be; therefore, appropriate parameters can be used in varying environments [29]. While, there are a wide range of parameters that provide the physical, chemical, nutrient and microbial aspects of water, among them, five are considered absolute fundamental parameters as illustrated in Fig 1.

## Methodology

The objective of this study was to monitor the quality of local bodies of water using five fundamental parameters of water namely pH, turbidity, TDS, DO and temperature. We built a system that could collect water samples as regular intervals and measure the condition of water. The design process was divided into several phases. The first phase was to evaluate off-shore and on-shore system that would greatly influence the design process. The second phase involved choosing sensors that would measure the fundamental water quality parameters. Subsequent stages include calculations for power consumption to select the appropriate battery capacity and solar panel, the hardware design, followed by development of firmware, and lastly, functional testing.

### Analysis of deployment methods

A field deployable water quality monitoring system could either be floating in water or secured on the coast. There are benefits and drawbacks to both. Sampling close to the coast could provide insights into the coastal environment and potential sources of pollution. It can also provide localized variations in water quality parameters compared to sampling away from the coast that can present a generalized condition of the water quality in the body of water [30]. Sampling away from the coast could also reduce the influence of coastal processes and potential contamination. This could be particularly helpful in studying widespread contamination and trends in water quality parameters, compared to sampling close to the coast [31, 32]. To study the general quality of the body of water, the ideal approach is to sample water away from the coast. Therefore, we evaluated different ways of deploying the sensors. Our first concept consisted of a system attached to a winch, which would only be lowered into the water to take measurements. The challenge with this approach was to find an appropriate place at every location to mount the heavy winch system along with heavy power demand involved with the use a winch system. Another concept was to build an enclosure that could be securely located on the shore with a long hose connected to a pump which would sample the water at regular intervals. Although, this was a credible system, which could also be cost effective, the concern was the variance in quality of water close to the shore compared to quality of water further away. Lastly, we settled on a flotation device, which would travel a few meters away from the shore and sample water. To do so, a system was developed which could house all the required components, that includes sensors, water sampling setup, and power management system.

### Sensor selection

Sensors are the backbone of the water quality monitoring system. There are a few factors to consider when choosing the right sensor, which are: measurement range, level of accuracy, sensitivity, stability, physical limitations, output signal type, and ease of operation [33]. Considering the stability and physical limitations of the sensor are crucial because a sensor that requires frequent calibration or maintenance for accurate functioning would rely on regular human intervention, which would make it less suitable for field-deployment.

DFRobot specializes in developing low-cost, sensitive, and field deployable sensors. In addition to being cost-effective, these were desirable due to their seamless compatibility with a variety of Arduino boards. Moreover, there are well established software drivers that are compatible with Arduino, which simplified the process of integrating the sensors with Arduino. Five sensors were incorporated into the final system. The sensors and their technical specifications are shown in Table 4. The DO sensor’s probe is galvanic and does not need polarization time. The sensor came with the signal converter module for easy interface to Arduino board through ADC. The turbidity sensor is able to detect suspended particles in water by measuring the light transmittance and scattering rate, which changes with the amount of TSS in water. TSS is direclty proportional to turbidity. The sensor comes with the signal converter module that connects to Arduino board through ADC. TDS sensor probe comprises of two needle like electrodes. The excitation source is an AC signal, which can effectively prevent the probe from polarization and prolong the life of sensor thereby achieving a stable output signal. The probe is encased in a waterproof housing with electrodes exposed, which enables the sensor to be immersed in water for long periods of time. The pH sensor probe houses a glass bulb electrode that maximizes the surface area of the sensor. This probe also comes with the signal converter module. The probe is laboratory grade and cannot stay immersed in water for prolonged periods of time. Temperature sensor probe houses DS18B20 thermistor chip that provides a 12-bit temperature reading. Since it has only one data line, it can easily be interfaced with Arduino boards without the need for a signal converter module.

**Table 4:** Five water quality sensors with technical specifications.

Sensor	Measurement Range	Resolution	Cost (USD)
pH	0 ∼10	± 0.1	$99.00
Temperature	-10°C ∼ 85°C	± 0.5°C	$7.50
Dissolved oxygen	0 ∼20 mg/L		$169.00
Total dissolved solids	0 ∼1000 ppm	± 10%	$11.80
Turbidity	0–4 NTU	± 7.5%	$9.90

### Power consumption and battery capacity calculation

The pumps are rated at 12V, 3A (peak), which is 36W of power. The electronics and sensors consume 500mA at 5V, totaling 2.5W. This leads to a total power requirement of the system = 38.5W.

However, the electronics side stays turned on continuously, but pumps are used only at intervals of 5 minutes. Therefore, the pumps are activated for a total of 12 times in 60 minutes (1 hour). If the pumps run for one minute every time they are tuned on, the calculation below displays the total power consumed by the pumps in an hour:

$$\begin{array}{c} (\frac{36W}{hr}÷\frac{60}{12})=\frac{7.2W}{hr} \end{array}$$ (1)

A maximum of 7.2W of power is consumed every hour by the pumps, and 2.5W of power consumed by electronics. Subsequently, 9.7W of power is consumed by the entire system every hour.

**Fig 3:**
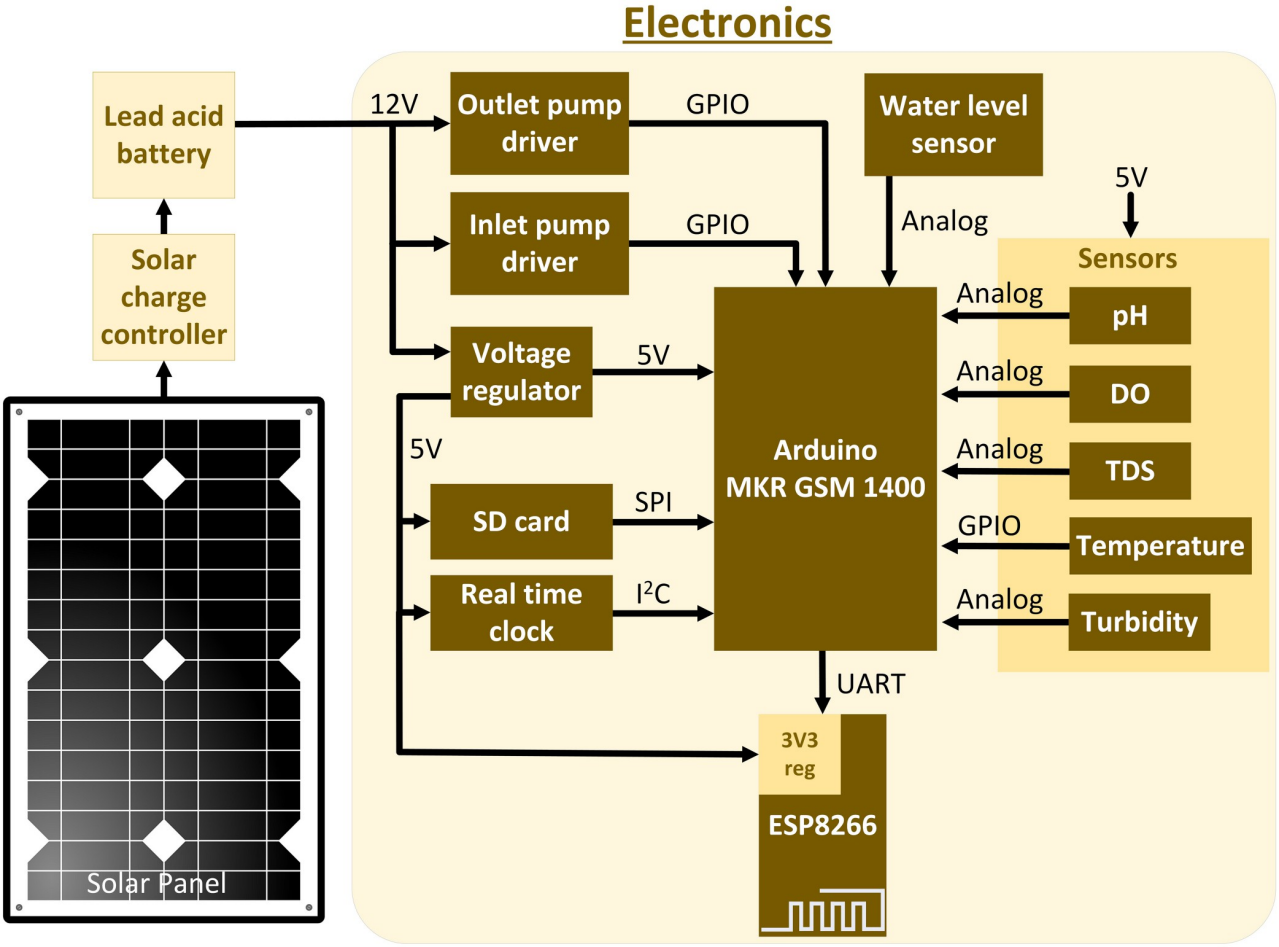
Overview of the hardware design.

In order to run the system overnight for approximately 10 hours, the required power is 9.7W x 10 = 97W. Therefore, for a 12V battery, 97W/12V = 8.08AH capacity will be required. Since, lead-acid batteries are not recommended to be discharged below 50% capacity, we will require at least 16.16AH battery. As a result, one 18AH or two 9AH or 10AH batteries will be sufficient to run the system for 10 hours without solar charge. The monocrystalline solar panel is rated at 80W with 4.55A of peak current generated on a bright sunny day. If the battery is depleted and charged using an 80W solar panel, it will take the battery approximately four hours to fully charge. Sealed lead-acid batteries or absorbent glass mat (AGM) deep cycle batteries are common choice for solar-powered systems. Since the pumps used in this system are rated at 12V, a 12V sealed lead-acid battery is appropriate and an economical choice. The Powertech solar charge controller provides up to 120W (12V x 10A) of power and connects in between the solar panel and the batteries to regulate the charge going into the batteries. It also ensures that the batteries are charged efficiently and prevents overcharging and over-discharging that prolongs the life of the battery. A block diagram of all the peripherals that connect to Arduino along with battery charging through solar charger is shown in Fig 3.

### System architecture

During the initial stages of system development, Arduino IoT cloud was incorporated, but we soon started running into issues with intermittent disconnection from cloud service. As an alternative, the text messaging service could have been used, but instead, external Wi-Fi module was employed to get quick system updates on a mobile device, which was thought to be cost-effective.

A water quality monitoring system has four primary components. First, there are sensors that measure various physical properties of the environment; as well as, 1100 GPH (Gallons per Hour) pumps, rated at 12V that were used for water sampling. Second, the use of electronics and programming that are responsible for enabling and collecting data from the sensors. The sensor could be transmitted to a cloud service and stored locally on some form of memory, such as SD card. Furthermore, keeping a track of date and time is also incorporated, using a Real-Time Clock (RTC), which is required to record date and time to capture timestamps of crucial events. Moreover, the system includes a solar power management system that ensures self-reliant operation of the system. And lastly, wireless communication module that establishes a connection between the system, the end-user, and the world. For a system that is deployed outdoors, weather conditions also need to be considered to ensure there is no sun or water damage.

**Fig 4:**
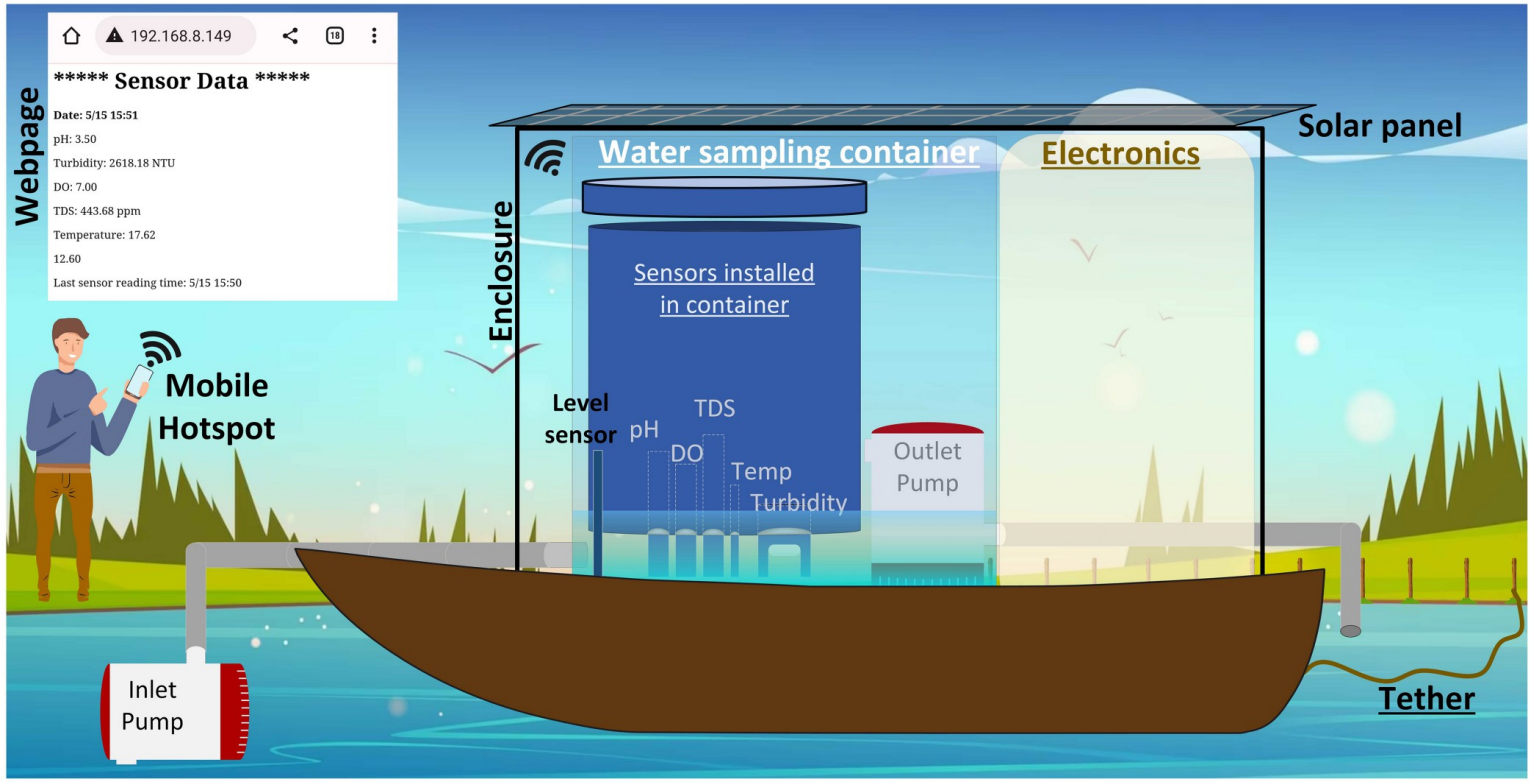
Illustration of the water quality monitoring system on a float boat.

The hardware architecture of the entire system is depicted in Fig 3 that shows the battery charging process through solar and the interaction of various peripherals with Arduino board. Among all the five sensors that we used, the pH sensor is the only laboratory grade sensor that was not designed for continuous immersion in water. Since, we could not continuously immerse the sensors in water, we adopted the water sampling method. The system uses Arduino MKR GSM 1400 board, which has a GSM module built-in. ARM cortex-M0 at its core, running at 48 MHz and provides enough analog and digital input/output pins to run the entire system with ease. The module supports 3G network connectivity which is capable of accessing the internet through GPRS data network as well send text messages to end user. It can also connect to Arduino IoT cloud or other cloud services. As previously mentioned, we did not use the GSM part of the board because initial tests suffered from frequent disconnection from Arduino IoT cloud, which was inefficient. This could be due to intermittent disconnection from the local 3G network. To build a robust system, an alternative communication method was chosen by using an ESP-8266 Wi-Fi module running a web server. The primary aim of a web page running on a server was to receive vital updates from the deployed system to ensure proper functioning if all the peripherals. This approach would also minimize the need for physical inspections, which would be challenging for a system positioned in water, a few meters away from the shore. Lastly, the inlet and outlet pumps were driven using logic level MOSFETs that use that supported a gate drive voltage as low as 1V. Fig 4 depicts an overview of the water quality monitoring system, showcasing a boat that is equipped with a water sampling container, electronics, and a solar panel positioned on top.

### Software architecture

Upon completion of the hardware design, programming the Arduino MKR board was subsequently completed. The drivers were programmed on Arduino IDE v2.0. An incremental software development style was adopted to program and test different modules of the system incrementally, which were later brought together as a whole. With this approach, the structure of the program could be controlled and through continuous testing, program crashes caused by software bugs could be prevented. The testing process began by programming and testing the sensors, to gain an understanding of the data they generated and interpret the received data to meaningful characteristics. After completing the programming, preliminary testing was conducted to verify both the hardware and the software.

**Fig 5:**
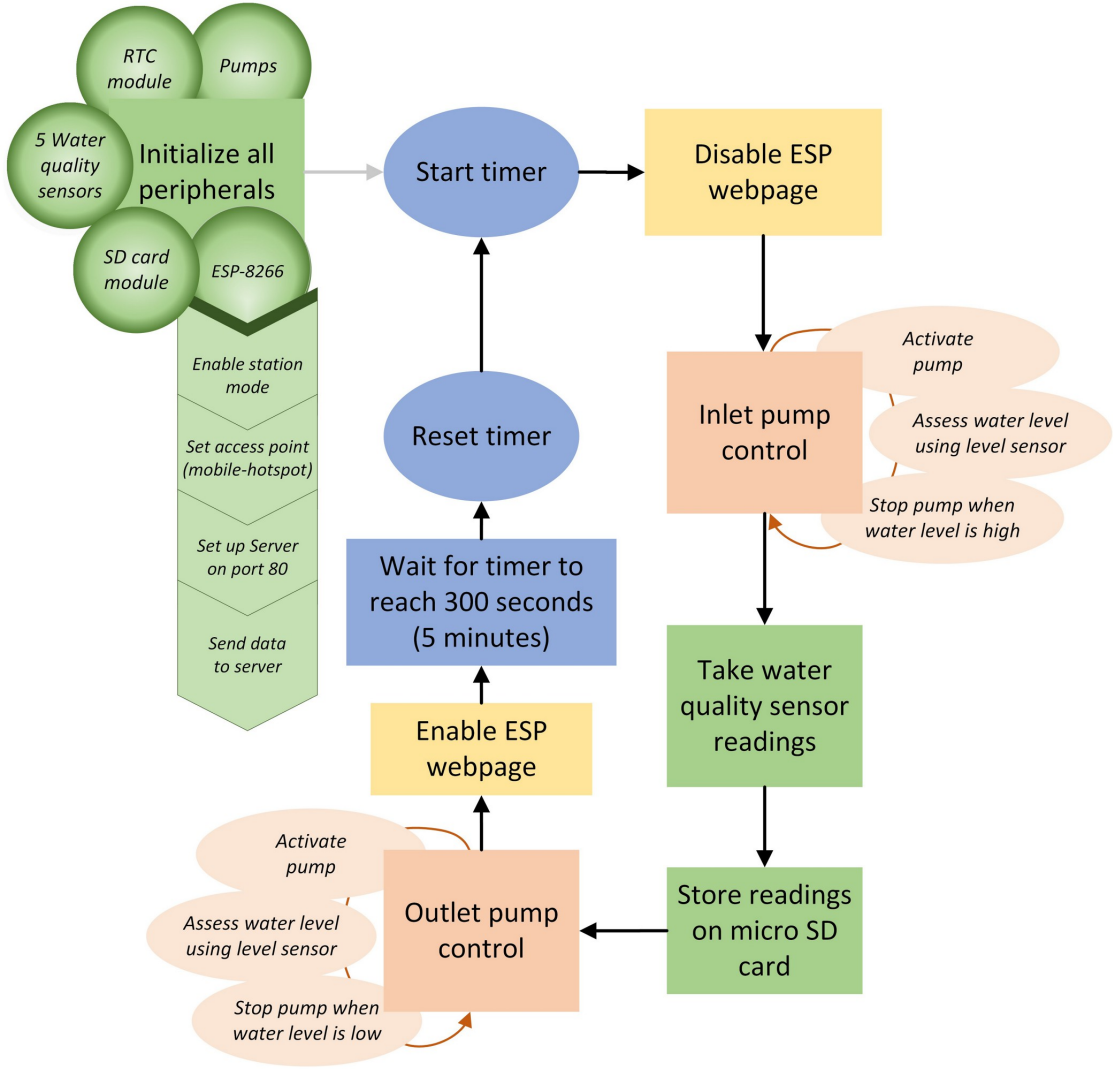
The flow chart illustrates the process of water sampling, sensor reading, and datalogging.

Fig 3 represents the system architecture that shows the flow of signals between Arduino and the peripherals and Fig 5 illustrates the flow of the firmware. Upon system startup, all the peripherals connected to Arduino are initialized. These include five water quality sensors configured as inputs to Arduino, pumps as outputs using PWM signal, micro-SD card module connected to the SPI bus, Real-Time Clock (RTC) connected to the I2C bus, and lastly, ESP-8266 Wi-Fi module that connected through UART. After the initialization process, the program enters a wait period of 10 minutes to allow the user enough time to deploy the system in water.

Thereafter, the program sequentially iterated through a series of instructions by first, activating the inlet pump only at a duty cycle 50% because of the pump’s rating of 1100 GPH. Turning on the pump at maximum power could pose a potential risk of flooding the water sampling container or even the boat. Continuously measured the water level was carried out by the water level sensor that turned off the inlet pump when the level sensor detected water level at its peak. With a fresh sample of water, the program continued to measure the parameters using the five water quality sensors. The data collected from the sensors was then stored on micro-SD card as a CSV file which it created on system startup. Upon successful storage of data, the program proceeded to activate the outlet pump at maximum power to flush the water out of the sampling container. Subsequently, the level sensor ensured that there was no residual water left behind. This prevented the new water sample from getting contaminated due to remnant of previous sample, thereby, ensuring the system was prepared for the next iteration of sampling and data collection which was set to 5 minutes.

The software also provides the user with the functionality of a web page that can be viewed on a mobile device. This was made possible with ESP-8266 Wi-Fi module which was configured as station mode that configures the device as a client, waiting to connect to an access point. The module was also configured as a server, accessible through port 80. A Wi-Fi hotspot was created on a mobile device that acts as an access point. The Wi-Fi module connects to the access point which then gets assigned an IP address in the form of 192.168.xxx.xxx. The user can access the web page that is running on the ESP server through this IP address. The information that was intended to be displayed on this web page was the current time that was directly accessed from the RTC, the time of last sensor reading, latest sensor data, battery voltage, as well as status updates, such as, malfunction of any of the sensors,pumps, or any issues with data storage on micro-SD card. Access to web page was disabled while the system collected water sample and gathered data from sensors, so that there is no conflict between ESP and the rest of the program operation.

It is worth noting that the process of activating pumps, reading sensor data, and data storage takes between 15-20 seconds to complete. The duration of this time period is subjective as it relies on the amount of water being pumped in, which depends on the state of the filter that is attached to the inlet pump. If the filter is dirty or clogged, the process of filling the water sampling container will be prolonged.

## Preliminary testing

**Fig 6:**
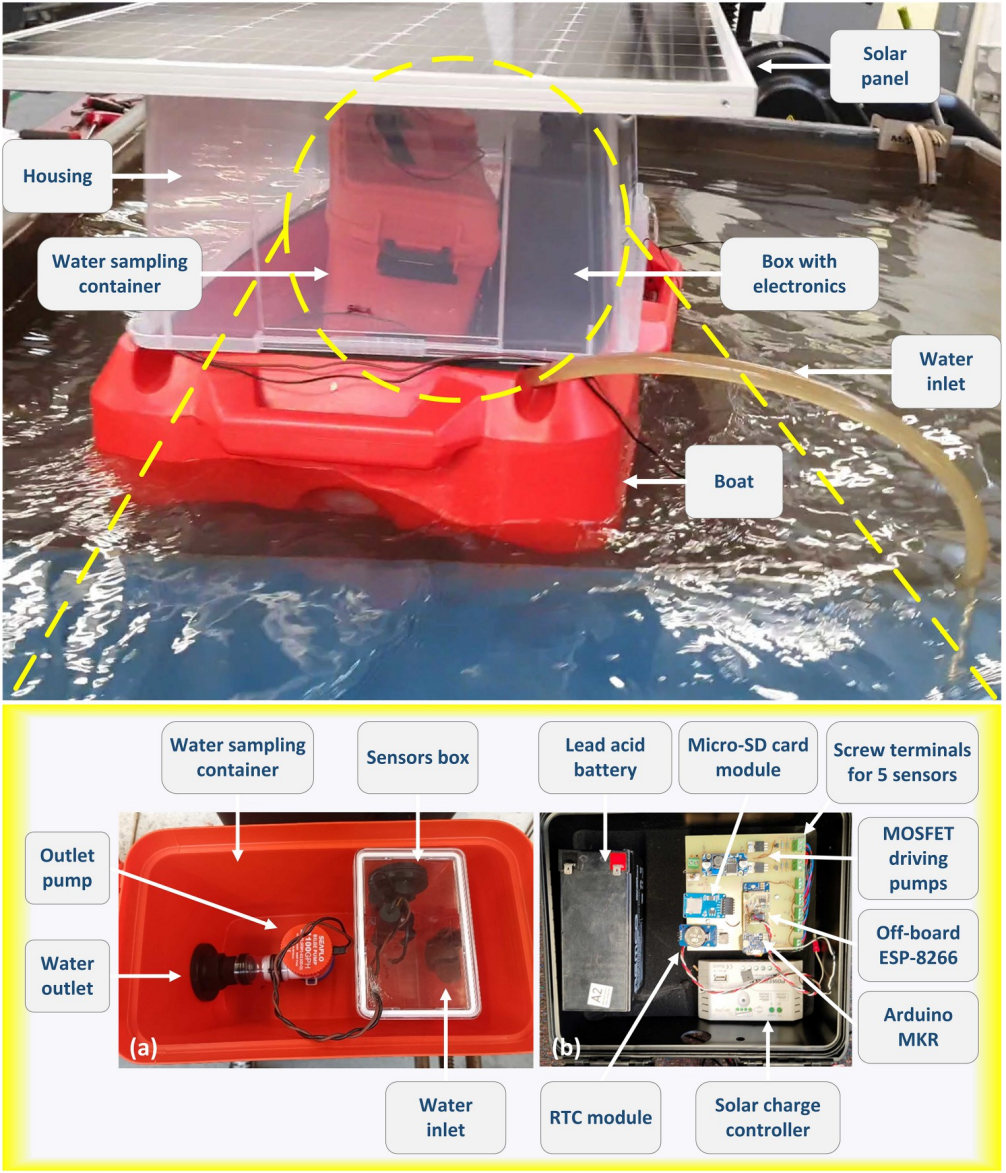
Initial testing of the system in pool the of water-jet cutter (Top); the system’s water sampling container and the electrodes (Bottom).

Preliminary testing was carried out in the water tank of the water-jet cutter as a preliminary test of the system as a whole. Since, this is a field-deployed system, the pumps, the sensing and the monitoring systems were thoroughly checked, to establish confidence of correct functioning of the system. This was achieved by running the system in the pool of the water-jet cutter at our mechanical engineering workshop. The water in the water-jet pool typically consists of small scraps or fragments of metal, primarily stainless steel and aluminium, along with grains of sand that resulted in a significant amount of suspended solids. A water-jet cutter pool was chosen to carry out initial testing of the system because it represented a worst-case scenario of the water conditions. This allowed us to test if the system could withstand the challenging water quality of the pool. Fig 6 displays the pool of the water-jet cutter with the boat floating, along with the box of electronics and container for water sampling, sheltered inside a transparent enclosure with the solar panel mounted on top.

## Results and discussion

**Fig 7:**
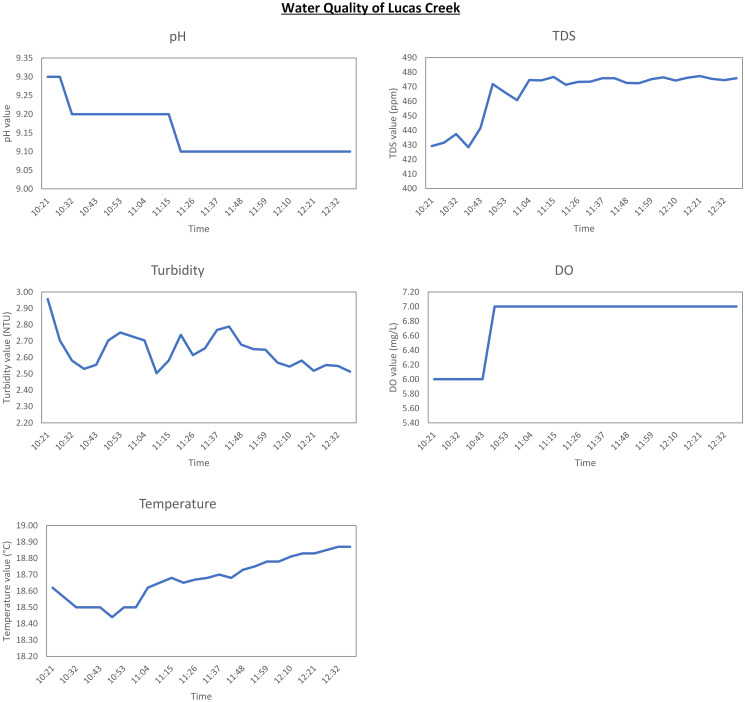
Results for Lucas creek.

**Fig 8:**
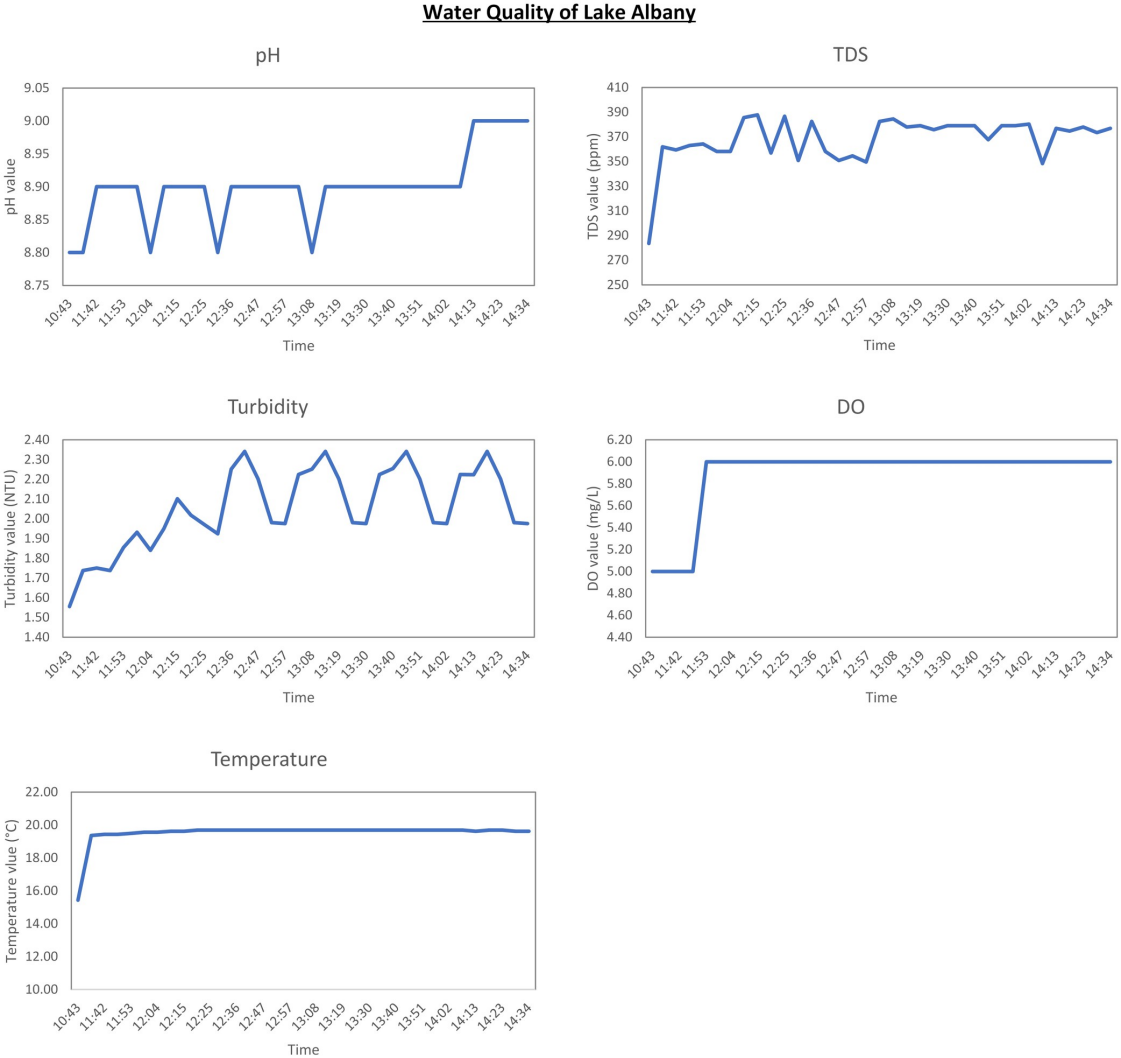
Results for Lake Albany.

**Fig 9:**
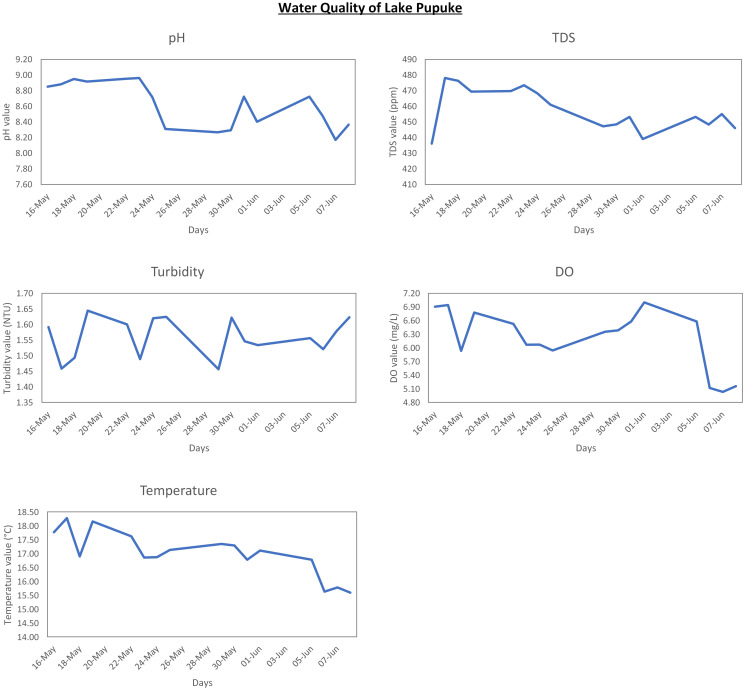
Results for Lake Pupuke.

The data gathered for this study provides valuable insights into the overall health of water around the local area. Figs 7–9 present data graphically from individual sensors for each location. As it can be seen from the graphs, the time period of the data gathered from Lucas creek and Lake Albany is quite short due to the water level being too low for any further tests. However, water level at Lake Pupuke was consistent which resulted in almost three weeks of data. Nevertheless, the results altogether yielded over three weeks of insightful data from three bodies of water.

Referring to the graphically depicted results for all the three locations, Lucas creek shows the highest turbidity level compared to the other two sites. Visually, Lucas creek had the cloudiest water as it can also be seen from Fig 2(1), compared to the clarity of water at Lake Pupuke that yielded low turbidity level. To thoroughly analyse the data, it is important to know the weather when the system was deployed at the two sites. At Lucas creek, it had rained a couple of hours prior to deploying the system, resulting in ample water, slowly flowing through the creek which was very muddy and polluted. On the other hand, it was a sunny day when data was gathered at Lake Albany. Looking at the results presented graphically in Figs 7 and 8, water at Lucas creek had higher level of alkalinity compared to Lake Albany, even though creek is a stream of water flowing continuously. Due to high cloudiness of water at the creek, the TDS value was higher compared to Lake Albany, peaking at 480 ppm and 390 ppm, respectively. This clearly states that creek water comprised of a higher level of dissolved solids. A similar trend can be seen from the high turbidity levels at the creek. The higher TDS and turbidity levels observed at the creek are evident due to the natural presence of muddy water. Furthermore, when examining the DO levels for both sites, it is evident that creek water, although it was turbid, carried more oxygen which can be seen from the high levels of dissolved oxygen of around 7 mg/L compared to 6 mg/L at Lake Albany. Regarding temperature, flowing water at the creek resulted in slightly lower temperatures compared to those observed at Lake Albany. After analyzing the quality of water at both sites, it is evident that despite the water being turbid, the oxygen levels remain relatively high compared to water at the lake.

Furthermore, analysing the results specifically for Lake Pupuke reveals a noticeable variation for a period of nearly three weeks from 16 May—8 June 2023. This is an ideal period because the season transitions from autumn to winter in New Zealand, and three weeks of data provided some insight on how the quality of water varies over time with seasonal changes as well as changes in weather conditions. A daily average was computed for all the sensors to present the mean values each day during the entire data collection period. These variations could either be influenced by weather or recreational activities that take place at the Lake. It rained between the periods 22-24 May which is indicated by a fall in temperature by 1.5°C, while pH value drops slightly from 8.8 to 8.2. A falling trend can also be seen from TDS data from 470 ppm to 450 ppm during this period. Between 1 June to 5 June, another noticeable event took place. During this period, the temperature dropped by approximately 0.5°C, while turbidity, TDS and pH data show an upward trend and DO demonstrated a declining trend. From the trends, it is clear that this is not a rain related event because it did not rain during this period.

Temperature and pH are inversely proportional to each other as it can be seen during periods 22-24 May, 24-26 May, and 30 May—1 June. Although, a contrary relationship is visible during 5-7 June period when the temperature and pH both have reduced. Analysing the overall temperature data, it can be seen that the temperature gradually dropped from 18.5°C to 16.5°C. This observation clearly indicates a seasonal change, transitioning to winter.

**Fig 10:**
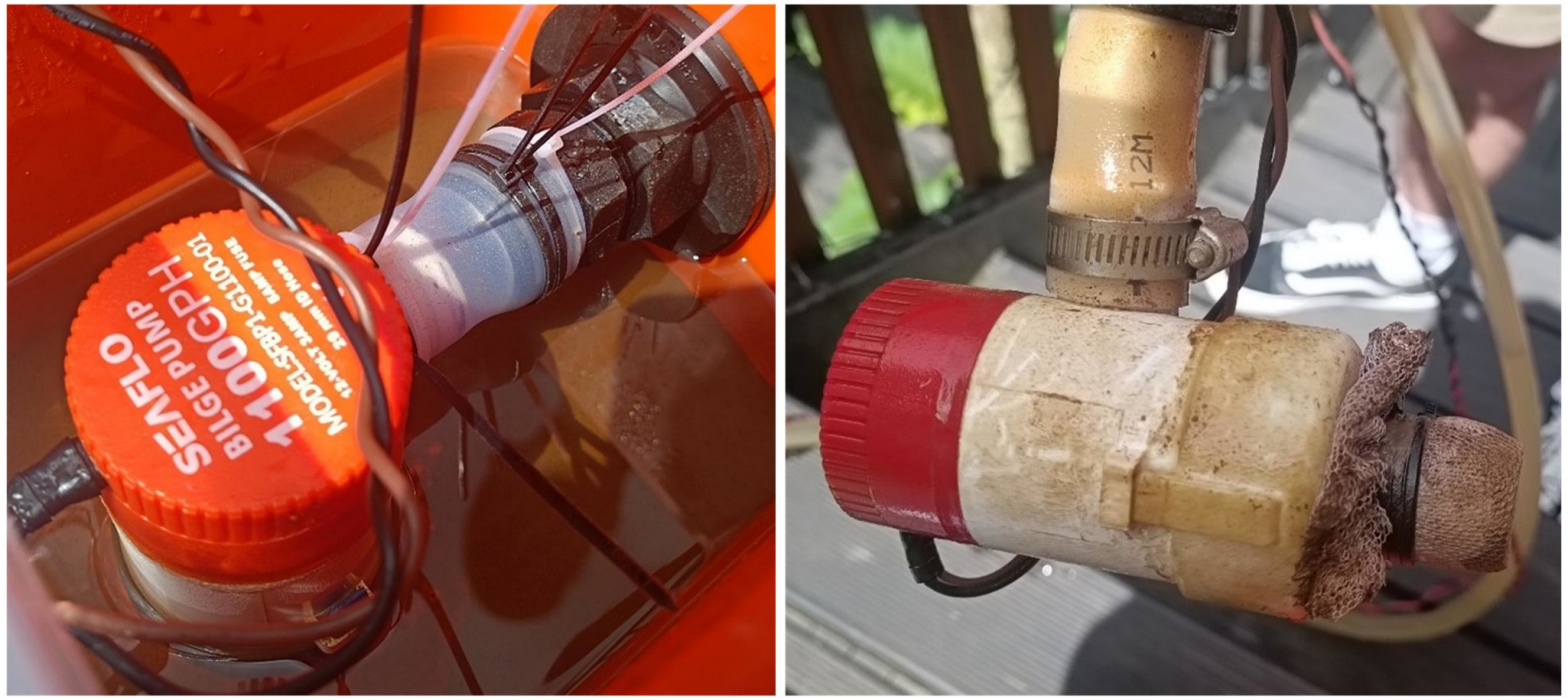
(left) sediment in water sampling tank, (right) small amount of dirt on inlet pump.

Some sensors were not recommended for continuous immersion in water. Therefore, a water sampling method was adopted, achieved by two pumps that circulated water in and out of a water sampling container. This container housed five water quality sensors as well as a water level sensor. The inlet pump was covered with a gauze filter to minimize the amount of dirt entering the container. Alternatively, an effort was made to utilize a filter mat, but it was found to be inadequate in capturing enough dirt. Although there was some dirt in the water sampling container and the inlet pump, found by the end of the data collection period which can be see in the Fig 10.

**Fig 11:**
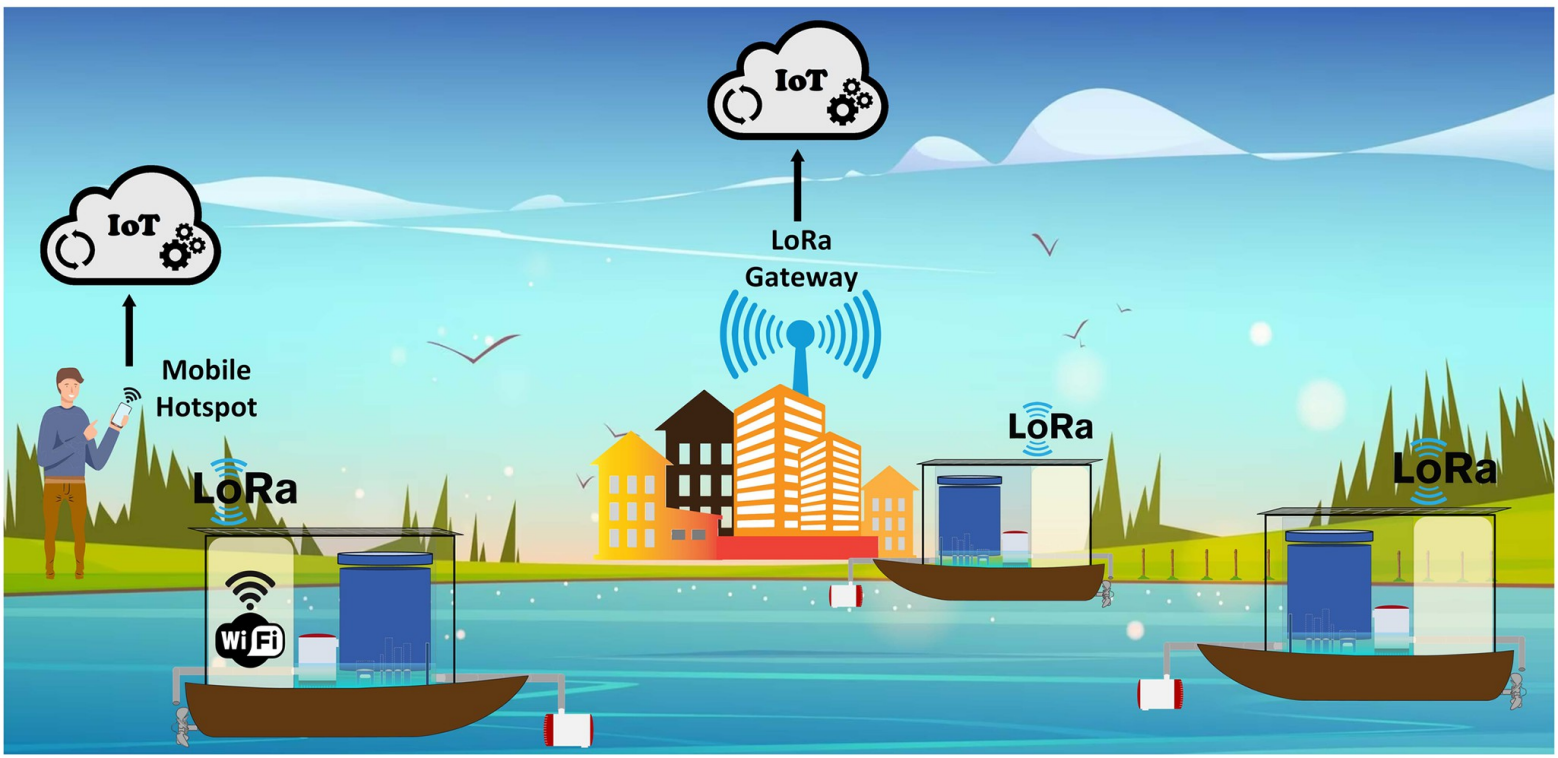
An extension of current work with more sensors and a few water quality systems distributed in water body, enabled with long range communications.

The system has been designed to be modular with scope of adding more sensors for further analysis such as monitoring and studying different types of nutrients or biological matter. This study can also be extended by building multiple systems that could be distributed around a lake or even a river. In order to cover a wider area, the boats could be equipped with propellers that will eliminate the need for tether and enable the boats to travel further away from the coast. Furthermore, all boats equipped with LoRa and low-cost Wi-Fi module can send data to IoT cloud service through LoRa gateway, if available or connect to a mobile hotspot that can collate data from all the LoRa nodes and send to IoT cloud through Wi-Fi using phone’s data. An illustration of such a system is shown in Fig 11. An alternative approach can be taken by using a single boat that would be pre-programmed to follow a path around the lake using a system called mission planner, commonly used with drones. This method would enable gathering of data from various locations within the same body of water to have a comprehensive understanding of water quality of the lake as a whole. Furthermore, using a GSM module, real-time weather updates can be received to correlate with the data to analyse weather related trends over time as well as send updates to the system such as new coordinates. Furthermore, the system is capable of adopting an Industry 4.0 paradigm, which is also known as the Fourth Industrial Revolution. It encompasses the use of automation, Big Data, Machine Learning (ML), combined with Artificial Intelligence (AI) and Internet of Things (IoT) [34]. In the age of Industry 4.0, systems are interconnected with information and communication, that results in scalability and autonomy.

## Conclusion

Regular water quality assessment is necessary to maintain clean and reliable water for everyone. However, existing water quality monitoring systems are either bulky and expensive or not easily field-deployable.

In this research, a water quality monitoring system consisting of low-cost sensors to measure the five basic water quality parameters (turbidity, total dissolved solids, temperature, pH, and dissolved oxygen) has been developed and tested. The system includes features like IoT technology, solar power, and the ability to float in fresh water like a small boat. Since the system floats in water, a water sampling technique has been adopted to avoid continuous contact of water with the sensors, which is detrimental, especially in low-cost sensors not designed for long-term immersion in water.

The data gathered from the sensors is stored locally on a micro-SD card, and communication with the system is established through an ESP-8266 module that operates as a web server. The web page running on the server is used to view current sensor values to ensure that the system is functioning as it should without the need for physical inspection. The web page also provides information such as battery voltage and status information, including any faults encountered by the sensors or pumps.

To test the efficacy of the system, data was gathered from Lake Albany and Lake Pupuke in the local area, providing insights into how the water quality of the two lakes compares. A brief study on the water quality of Lucas Creek, with slow-moving water, was also performed. The water in this creek is considered somewhat fresh. Fast-moving rivers or oceans were excluded from the study because the variation in water quality would be too rapid for the low-cost sensors to handle.

From the results, particularly for Lake Pupuke, it can be observed that the levels of all the five parameters are quite stable with a variation of ±10%, which was expected from a lake as it is not a moving body of water. This confirms that the developed system can be reliably used for further studies. The system operated in water bodies for several days to weeks and thus proved to be robust and autonomous in effectively monitoring the quality of water in real-time. It also carries the potential of adding more sensors and employing the Industry 4.0 paradigm to predict variations in water quality.
